# Real-world skin and dermatology-specific quality-of-life outcomes and a nomogram to predict skin response to secukinumab in Chinese psoriatic arthritis patients: a multicenter retrospective cohort study

**DOI:** 10.3389/fmed.2026.1773701

**Published:** 2026-04-16

**Authors:** Lihua Zhang, Zhenzhen Xu, Jian Gong, Xiaohua Tao, Fengming Hu

**Affiliations:** 1 Dermatology Hospital of Jiangxi Province, Nanchang, China; 2 The Affiliated Dermatology Hospital of Nanchang University, Nanchang, China; 3 Jiangxi University of Traditional Chinese Medicine, Nanchang, China; 4 Candidate Branch of National Clinical Research Center for Skin Diseases, Nanchang, China; 5 National Clinical Research Center for Skin and Immune Disease, Beijing, China

**Keywords:** DLQI, nomogram, PASI, prediction model, psoriasis, psoriatic arthritis, real-world study, secukinumab

## Abstract

**Background:**

Real-world data on secukinumab in Chinese psoriatic arthritis (PsA) patients remain limited, particularly regarding short-term cutaneous response, dermatology-specific quality of life, and predictors of treatment response.

**Methods:**

A retrospective cohort study was performed using 446 PsA patients diagnosed based on the Classification Criteria for Psoriatic Arthritis (CASPAR) criteria and treated with secukinumab for >12 weeks. Skin severity and quality of life were assessed using the Psoriasis Area and Severity Index (PASI), Body Surface Area (BSA), Investigator’s Global Assessment (IGA), and Dermatology Life Quality Index (DLQI) at baseline and after treatment. Treatment-related adverse events (TRAEs) were graded according to CTCAE v5.0. A predictive nomogram was constructed using stepwise multivariate regression and validated using the receiver operating characteristic (ROC) analysis.

**Results:**

The cohort of 446 PsA patients (mean age 47.7 years; 65.2% men) exhibited significant improvements across all domains. Post-treatment PASI decreased from 14.2 ± 14.4 to 3.98 ± 8.84 (*p* < 0.001), and DLQI improved from 11.9 ± 7.44 to 4.41 ± 5.63 (*p* < 0.001). The correlation between PASI and DLQI significantly strengthened post-treatment (*R*^2^ increased from 0.34 to 0.52, Δ*R*^2^ = +0.18; *p* < 0.001), accompanied by a steeper regression slope (0.3479 vs. 0.2229). Post-treatment objective-subjective outcome intercorrelations were robust (IGA-PASI *r* = 0.86, PASI-DLQI *r* = 0.62, all *p* < 0.001). A predictive nomogram incorporating five baseline variables — PASI score, disease duration, patient age, baseline BSA, and body mass index (BMI) — was developed and internally validated (AUC = 0.764, 95% CI: 0.712–0.816) for predicting cutaneous treatment response. Higher baseline PASI scores, longer disease duration, greater baseline BSA, older age, and lower BMI were associated with higher predicted probability of achieving treatment response.

**Conclusion:**

In this real-world cohort of Chinese PsA patients, secukinumab demonstrates robust clinical short-term efficacy in skin severity and dermatology-specific quality of life, with a favorable short-term safety profile. The predictive nomogram may assist in estimating short-term cutaneous response, although external validation is required. However, these findings are specific to PsA patients and should not be extrapolated to psoriasis populations without musculoskeletal involvement, given the additional disease-burden dimensions in PsA that influence quality-of-life outcomes.

## 1 Introduction

Psoriatic arthritis (PsA) is a complex, chronic, immune-mediated inflammatory disease that affects approximately 0.5–1% of the global population (1). Characterized by high clinical heterogeneity, PsA manifests through peripheral arthritis, enthesitis, dactylitis, axial involvement, and cutaneous psoriasis (2). Beyond physical joint destruction and functional impairment, PsA imposes a profound psychosocial burden on patients; epidemiological studies indicate that up to 42% of patients suffer from comorbid depression and 35% from anxiety, leading to a significant reduction in quality of life (QOL) that often rivals or exceeds that of other major systemic diseases (3, 4). The PsA pathogenesis involves a sophisticated interplay between genetic susceptibility (such as human leukocyte antigen; HLA-B27, interleukin-23R; and IL-23R polymorphisms), environmental triggers, and dysregulated innate and adaptive immune responses (5, 6). The core of this immunopathological framework is the IL-23/Th17 signaling axis. Under inflammatory conditions, IL-23 promotes the differentiation of naive CD4^+^ T cells into Th17 cells and stimulates the expansion of IL-17-producing γδ T cells and innate lymphoid cells (ILCs) (7). The resultant IL-17A overexpression acts as a master orchestrator of tissue-specific inflammation, driving keratinocyte hyperproliferation in the skin, synovial fibroblast activation in the joints, and neutrophilic infiltration, also contributing to systemic manifestations such as intestinal barrier dysfunction (8, 9).

The therapeutic landscape for PsA has evolved significantly from traditional disease-modifying antirheumatic drugs (DMARDs) to biological agents. While tumor necrosis factor-alpha inhibitors (TNFi) and IL-12/23 inhibitors have demonstrated substantial efficacy, concerns regarding secondary loss of response, and risks of serious infections or tuberculosis reactivation persist (10, 11). Secukinumab, a fully human monoclonal antibody that selectively neutralizes IL-17A, has emerged as a high-precision therapy. Unlike broader immunosuppressants, secukinumab preserves Th1-mediated immunity and regulatory T-cell (Treg) function, potentially offering a superior efficacy-to-safety profile (12). Although the landmark Phase III FUTURE trials established the clinical efficacy of secukinumab, several critical knowledge gaps remain in the real-world setting.

Firstly, evidence from randomized controlled trials (RCTs) is often constrained by stringent inclusion/exclusion criteria, leaving the long-term effectiveness of secukinumab in diverse, real-world patient populations (including those with multiple comorbidities) incompletely characterized (13). Secondly, while objective measures such as the PASI and subjective measures like the DLQI are standard, their dynamic interrelationship during IL-17A inhibition has not been systematically integrated. This limits the understanding of the degree to which immunological clearance of the skin translates into psychosocial recovery.

To address these limitations, a large-scale, real-world observational cohort study (*n* = 446) was conducted to evaluate the multidimensional efficacy and safety of secukinumab in PsA. This study specifically aimed to: (i) quantify the synchronized dynamic relationship between PASI and DLQI as a proxy for integrated immunological benefit; (ii) develop and validate a robust clinico-biological predictive model for treatment response; and (iii) provide a comprehensive safety profile assessment in a real-world clinical context.

## 2 Materials and methods

### 2.1 Study design and setting

This retrospective cohort study was conducted using data from the China Psoriasis Standardized Diagnosis and Treatment Center database, a multicenter registry that prospectively collects comprehensive clinical information from patients with psoriasis and psoriatic arthritis (PsA) receiving standardized care at participating dermatology centers across China. The China Psoriasis Standardized Diagnosis and Treatment Center database is a dermatology-based registry that systematically captures cutaneous disease severity measures and patient-reported quality-of-life outcomes. Musculoskeletal outcome measures specific to PsA (e.g., ACR response criteria, tender/swollen joint counts, enthesitis and dactylitis scores) are not routinely collected within this registry framework. The study period extended from June 2020 to September 2024.

### 2.2 Ethical considerations

This study was conducted in accordance with the ethical principles outlined in the Declaration of Helsinki and the International Conference on Harmonisation Guidelines for Good Clinical Practice. The study protocol was reviewed and approved by the Institutional Ethics Committee of the Dermatology Hospital of Jiangxi Province (Approval No. KY2026-02-01). All participants provided written informed consent for the collection and use of their anonymized clinical data for research purposes at the time of enrollment in the registry.

### 2.3 Study population

#### 2.3.1 Patient selection

Among 25,561 psoriasis patients treated with biologic agents during the study period, 558 patients with PsA who received secukinumab therapy and had complete follow-up records were initially identified. After applying predefined exclusion criteria, 112 patients were excluded due to incomplete baseline data (*n* = 67), undocumented medication history (*n* = 31), and data entry errors (*n* = 14), resulting in a final analytical cohort of 446 patients.

#### 2.3.2 Inclusion criteria

Patients were eligible for inclusion if they met all of the following criteria: (1) age ≥18 years at treatment initiation; (2) diagnosis of PsA according to the Classification Criteria for Psoriatic Arthritis (CASPAR) (14); (3) treatment with secukinumab at the approved dosing regimen (300 mg administered subcutaneously at weeks 0–4 as loading doses, followed by 300 mg every 4 weeks as maintenance therapy); and (4) availability of complete baseline and follow-up clinical assessment data.

#### 2.3.3 Exclusion criteria

Patients were excluded if they met any of the following criteria: (1) missing critical baseline clinical information; (2) data inconsistencies or errors identified during quality control verification; or (3) undocumented prior medication history precluding accurate characterization of treatment history.

### 2.4 Data collection

#### 2.4.1 Demographic variables

Baseline demographic data extracted from the registry included: gender, age at treatment initiation, marital status, and body mass index (BMI). BMI was calculated as weight in kilograms divided by the square of height in meters (kg/m^2^) and categorized according to the World Health Organization criteria adapted for Asian populations.

#### 2.4.2 Disease-related variables

Clinical variables collected at baseline included: disease duration, family history of psoriasis (defined as presence of psoriasis in first-degree relatives), and prior treatment history. Disease duration was defined as the interval (in years) from the initial onset of psoriasis symptoms to secukinumab initiation and was categorized into three groups, namely <10 years, 10–20 years, and >20 years. Prior biologic therapy was documented, including the specific biologic agents administered before secukinumab initiation. Patient-reported treatment satisfaction with prior therapies was assessed using a 5-point Likert scale with the following response categories: very satisfied, somewhat satisfied, satisfied, dissatisfied, and very dissatisfied.

### 2.5 Clinical assessments

Disease severity and treatment response were evaluated using validated instruments at baseline and at the post-treatment evaluation visit (week 12–16), as detailed in the following section.

#### 2.5.1 Psoriasis Area and Severity Index (PASI)

The PASI is a validated composite measure that quantifies both the severity and extent of psoriatic skin involvement. The assessment evaluates three clinical characteristics—erythema (E), induration (I), and desquamation (D)—across four anatomical regions: head and neck, upper extremities, trunk, and lower extremities. Each characteristic is scored on a 5-point scale ranging from 0 (absent) to 4 (severe). Regional scores were calculated by multiplying the sum of severity scores (E + I + D) by the region-specific body surface area weighting factor (head/neck: 0.1; upper extremities: 0.2; trunk: 0.3; and lower extremities: 0.4) and the affected area score (0–6 points, reflecting the proportion of each region involved). The total PASI score represents the cumulative sum of all regional scores, with possible values ranging from 0 to 72, where higher scores indicate more severe disease.

#### 2.5.2 Body surface area (BSA)

The percentage of total body surface area (BSA) affected by psoriasis was estimated using the Chinese Rule of Nines methodology, which assigns the following proportions to anatomical regions: head and neck (9%), upper extremities (18%), trunk (27%), and lower extremities (46%). The sum of affected areas across all regions yielded the total BSA involvement expressed as a percentage.

#### 2.5.3 Investigator’s Global Assessment (IGA)

The IGA provides an overall assessment of current psoriasis severity based on the investigator’s clinical judgment. A 5-point ordinal scale was employed: 0 = clear (no signs of psoriasis), 1 = almost clear (minimal evidence), 2 = mild (limited disease), 3 = moderate (clearly evident disease), and 4 = severe (extensive disease). All assessments were performed by board-certified dermatologists trained in standardized evaluation protocols.

#### 2.5.4 Dermatology Life Quality Index (DLQI)

The DLQI is a validated, dermatology-specific patient-reported outcome measure that assesses the impact of skin disease on health-related quality of life over the preceding week. The instrument comprises 10 items addressing six domains: symptoms and feelings, daily activities, leisure, work and school, personal relationships, and treatment burden. Each item is scored from 0 (not at all/not relevant) to 3 (very much), yielding total scores ranging from 0 (no impairment) to 30 (maximum impairment).

DLQI scores were interpreted according to established banding categories: 0–1 = no effect on the patient’s life, 2–5 = small effect, 6–10 = moderate effect, 11–20 = very large effect, and 21–30 = extremely large effect on quality of life. A score of ≤1 was considered to indicate no clinically meaningful impact, while scores ≥2 were interpreted as reflecting measurable impairment warranting clinical attention.

### 2.6 Outcome measures

#### 2.6.1 Efficacy outcomes

The primary efficacy outcomes were absolute changes from baseline to post-treatment assessment in PASI, BSA, IGA, and DLQI scores (15). PASI ratio was defined as (bPASI−aPASI)/bPASI×100% .

The treatment efficacy index was derived from the PASI ratio, and the efficacy outcomes were classified into two categories, namely efficacy response and non-response (≤30% improvement).

#### 2.6.2 Safety outcomes

Treatment-related adverse events (TRAEs) occurring during the study period were collected from medical records. Adverse events were graded according to the Common Terminology Criteria for Adverse Events (CTCAE) version 5.0. Serious adverse events (SAEs) were also recorded. Adverse event data were collected through retrospective review of clinical documentation. Only events explicitly documented by the treating physician and judged to be related to secukinumab therapy were classified as TRAEs. No standardized active solicitation protocol was employed. This passive ascertainment approach is recognized as likely to underestimate true adverse event incidence relative to the active surveillance mandated in prospective clinical trials.

### 2.7 Statistical analysis

All statistical analyses were performed using R software (version 4.1.2; R Foundation for Statistical Computing, Vienna, Austria). A two-sided *p*-value of <0.05 was considered statistically significant for all analyses.

#### 2.7.1 Descriptive statistics

Continuous variables were assessed for normality using the Shapiro–Wilk test and visual inspection of histograms. Normally distributed continuous variables were expressed as mean ± standard deviation (SD), while non-normally distributed variables were presented as median and interquartile range (IQR). Categorical variables were summarized as frequencies and percentages.

#### 2.7.2 Correlation analysis

Pairwise correlations among continuous clinical variables (age, disease duration, BMI, PASI, BSA, IGA, and DLQI) were assessed using the Pearson’s correlation coefficient for normally distributed data or the Spearman’s rank correlation coefficient for non-normally distributed or ordinal data. Correlation strength was interpreted as: |*r*| < 0.3 = weak, 0.3 ≤ |*r*| < 0.5 = moderate, 0.5 ≤ |*r*| < 0.7 = strong, and |*r*| ≥ 0.7 = very strong (7). A correlation matrix heatmap was generated to visualize the interrelationships among multiple variables.

#### 2.7.3 Pre-post treatment comparisons

Within-group differences in BSA, PASI, IGA, and DLQI scores before and after treatment were evaluated using the paired Student’s *t*-tests for normally distributed data or the Wilcoxon signed-rank tests for non-parametric data. Effect sizes were calculated using the Cohen’s *d* for normally distributed paired comparisons.

#### 2.7.4 Regression analysis

The longitudinal relationship between DLQI and PASI was analyzed using the simple linear regression at baseline and post-treatment timepoints. Scatterplots with superimposed regression lines and 95% confidence intervals were generated to visualize these relationships. Changes in regression coefficients and coefficients of determination (*R*^2^) between baseline and post-treatment were examined to characterize the evolution of the PASI–DLQI relationship following treatment.

#### 2.7.5 Predictive model development

A multivariate regression model was developed to predict secukinumab treatment efficacy. Candidate predictor variables included baseline clinical characteristics: PASI score, disease duration, age, gender, BMI, BSA, IGA, DLQI, family history, prior biologic exposure, and pretreatment satisfaction. Variable selection was performed using forward stepwise regression based on the Akaike Information Criterion (AIC), with entry criterion *p* < 0.05 and removal criterion *p* > 0.10.

A nomogram was constructed to provide a graphical representation of the final predictive model, facilitating clinical application by allowing calculation of predicted treatment response probabilities based on individual patient characteristics (16). Each predictor variable was assigned a point value proportional to its regression coefficient, with the total points corresponding to the predicted probability of treatment response.

#### 2.7.6 Model validation

Internal validation of the predictive model was performed using 10-fold cross-validation to assess model stability and reduce overfitting. Discriminative performance was evaluated using the area under the receiver operating characteristic curve (AUC–ROC), with interpretation as follows: AUC 0.5–0.6 = fail, 0.6–0.7 = poor, 0.7–0.8 = fair, 0.8–0.9 = good, and >0.9 = excellent (9). Model calibration was assessed by comparing predicted probabilities with the observed outcomes across risk strata, visualized using calibration plots.

## 3 Results

### 3.1 Baseline characteristics of the study population

The baseline demographic and clinical characteristics of the 446 PsA patients included in this study are summarized in Table 1. The cohort demonstrated a male predominance (65.2%, *n* = 291) with a mean age of 47.7 ± 12.8 years. The mean BMI was 25.4 ± 4.2 kg/m^2^, and the majority of patients (81.0%, *n* = 361) reported no family history of psoriasis, while 19.0% (*n* = 85) had a positive family history in first-degree relatives. Regarding marital status, 86.1% (*n* = 384) of patients were married, and 13.9% (*n* = 62) were unmarried. The mean disease duration was 13.4 ± 10.6 years. Prior treatment history revealed that 67.5% (*n* = 301) of patients were biologic-naïve (first-time secukinumab users), while 32.5% (*n* = 145) had received prior biologic therapy. Pretreatment satisfaction with prior therapies varied considerably: 9.19% (*n* = 41) reported being “very satisfied,” 31.6% (*n* = 141) were “somewhat satisfied,” 19.3% (*n* = 86) were “satisfied,” 26.0% (*n* = 116) expressed dissatisfaction, and 7.17% (*n* = 32) were “very dissatisfied.” Satisfaction status was unknown for 6.73% (*n* = 30) of patients.

**Table 1:** Baseline demographic and clinical characteristics of the study population.

Characteristic	Value (*N* = 446)
Demographics	
Age, years, mean ± SD	47.7 ± 12.8
Male, gender, *n* (%)	291 (65.2)
BMI, kg/m^2^, mean ± SD	25.4 ± 4.2
Married, *n* (%)	384 (86.1)
Disease characteristics	
Disease duration, years, mean ± SD	13.4 ± 10.6
Family history of psoriasis, *n* (%)	85 (19.0)
Treatment history	
Biologic-naïve, *n* (%)	301 (67.5)
Prior biologic exposure, *n* (%)	145 (32.5)
Pretreatment satisfaction, *n* (%)	
Very satisfied	41 (9.19)
Somewhat satisfied	141 (31.6)
Satisfied	86 (19.3)
Dissatisfied	116 (26.0)
Very dissatisfied	32 (7.17)
Unknown	30 (6.73)
Baseline disease severity	
PASI, mean ± SD	14.2 ± 14.4
BSA, %, mean ± SD	25.7 ± 25.9
IGA, mean ± SD	2.8 ± 1.1
DLQI, mean ± SD	11.9 ± 7.44
Post-treatment outcomes	
PASI, mean ± SD	3.98 ± 8.84
BSA, %, mean ± SD	8.26 ± 15.2
DLQI, mean ± SD	4.41 ± 5.63
PASI ratio, mean ± SD	0.61 ± 0.68

BMI, body mass index; BSA, body surface area; DLQI, Dermatology Life Quality Index; IGA, Investigator’s Global Assessment; PASI, Psoriasis Area and Severity Index; SD, standard deviation.

At baseline, disease severity indices indicated moderate-to-severe disease: mean PASI was 14.2 ± 14.4, mean BSA involvement was 25.7 ± 25.9%, mean IGA was 2.8 ± 1.1, and mean DLQI was 11.9 ± 7.44, reflecting a substantial impact on health-related quality of life.

### 3.2 Efficacy of secukinumab treatment

#### 3.2.1 Clinical efficacy outcomes

**Figure 1:**
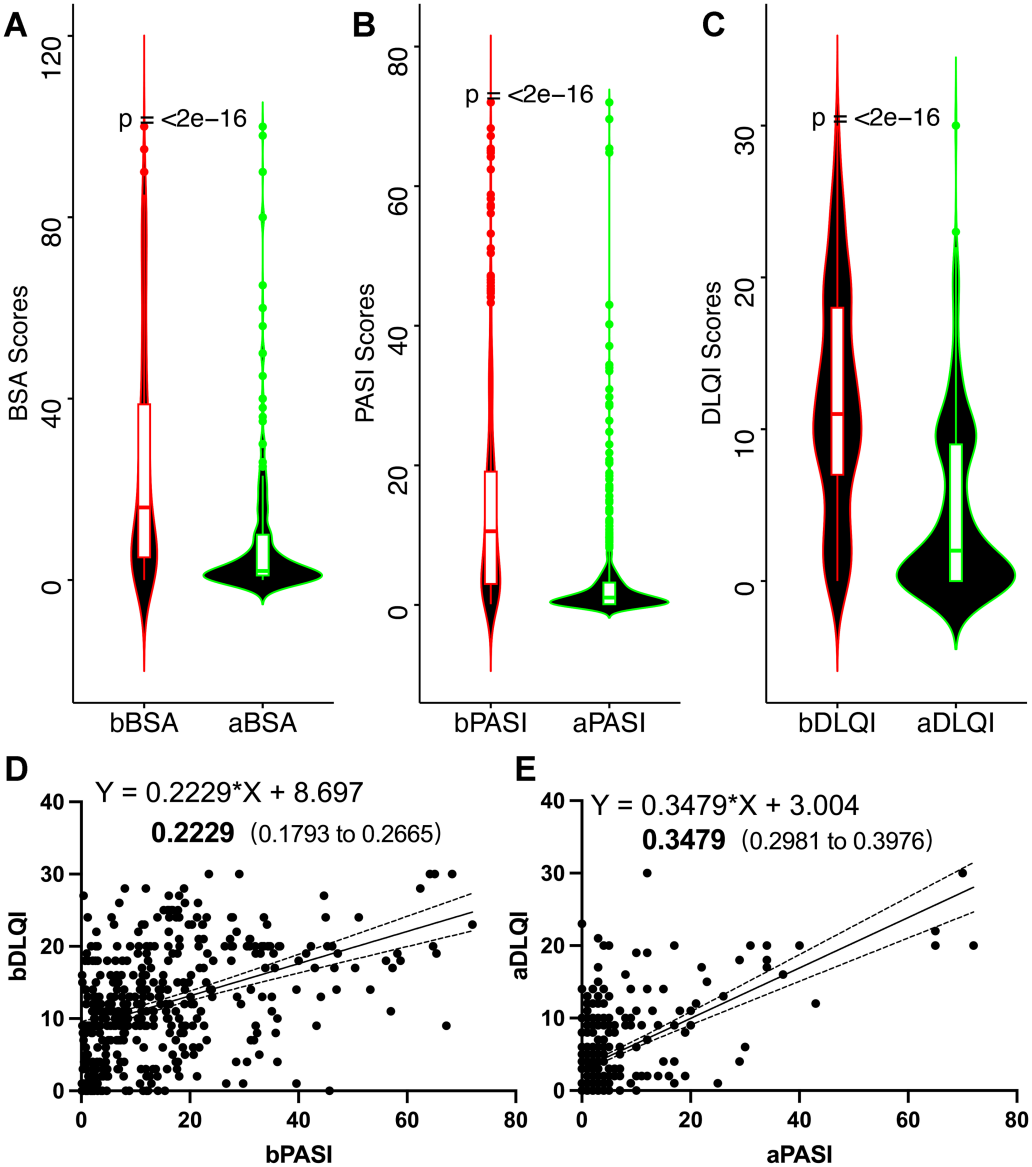
Clinical efficacy of secukinumab treatment in psoriatic arthritis. **(A)** Body surface area (BSA) involvement at baseline and post-treatment. **(B)** Psoriasis Area And Severity Index (PASI) scores at baseline and post-treatment. **(C)** Dermatology Life Quality Index (DLQI) scores at baseline and post-treatment. **(D)** Linear regression analysis of the relationship between PASI and DLQI at baseline. **(E)** Linear regression analysis of the relationship between PASI and DLQI post-treatment. Data are presented as mean ± SD. Statistical comparisons were performed using the paired *t*-tests. ***p < 0.001. bBSA, baseline BSA; aBSA, post-treatment BSA; bPASI, baseline PASI; aPASI, post-treatment PASI; bDLQI, baseline DLQI; aDLQI, post-treatment DLQI.

Secukinumab treatment was associated with statistically significant and clinically meaningful improvements across all evaluated disease severity indices and patient-reported outcomes (Figures 1A–C). BSA involvement demonstrated a substantial reduction from baseline to post-treatment assessment (25.7 ± 25.9% vs. 8.26 ± 15.2%, respectively; mean change: −17.4%; *p* < 0.001; Figure 1A), representing a 67.9% relative reduction in affected skin surface area. PASI scores decreased significantly from baseline to post-treatment (14.2 ± 14.4 vs. 3.98 ± 8.84, respectively; mean change: −10.2; *p* < 0.001; Figure 1B), corresponding to a 72.0% relative improvement. The mean PASI ratio, reflecting proportional improvement from baseline, was 0.61 ± 0.68. Patient-reported quality of life, as assessed by the DLQI, improved markedly following secukinumab treatment (11.9 ± 7.44 vs. 4.41 ± 5.63, respectively; mean change: −7.5; *p* < 0.001; Figure 1C).

#### 3.2.2 Dynamic relationship between clinical severity and quality of life

A key finding of this study was the characterization of how the relationship between objective disease severity (PASI) and subjective quality of life impairment (DLQI) evolved following treatment. At baseline, linear regression analysis revealed a moderate positive correlation between PASI and DLQI (*Y* = 0.2229X + 8.697, *R*^2^ = 0.34, *p* < 0.001; Figure 1D), indicating that approximately 34% of the variance in baseline quality of life impairment was explained by the disease severity. Following secukinumab treatment, this relationship strengthened substantially, with a steeper regression slope and enhanced coefficient of determination (*Y* = 0.3479X + 3.004, *R*^2^ = 0.52, *p* < 0.001; Figure 1E). The increase in *R*^2^ from 0.34 to 0.52 indicates that post-treatment, 52% of the variance in quality of life was explained by residual disease severity—a 53% relative increase in explanatory power.

This enhanced concordance between clinical improvement and quality of life gains suggests that effective treatment aligns both the objective and subjective outcomes. The steeper post-treatment regression slope (0.3479 vs. 0.2229) further indicates that following effective therapy, each unit change in PASI is associated with a larger corresponding change in DLQI, suggesting heightened patient sensitivity to residual disease burden.

### 3.3 Safety profile of secukinumab

Secukinumab demonstrated a favorable safety profile in this real-world PsA cohort. Treatment-related adverse events (TRAEs) were reported in only 4 of 446 patients (0.90%), all of which were mild to moderate in severity (Grade 1–2 according to CTCAE v5.0). The reported TRAEs comprised two mucocutaneous reactions—pharyngeal edema (*n* = 1) and self-limiting urticaria (*n* = 1)—and two gastrointestinal events—abdominal distension (*n* = 1) and intermittent nausea (*n* = 1). All adverse events were transient in nature and resolved spontaneously without requiring dose modification, treatment interruption, or discontinuation. Notably, no severe adverse events (Grade ≥3), serious adverse events (SAEs), opportunistic infections, inflammatory bowel disease, major adverse cardiovascular events, malignancies, or deaths were reported during the study period. These findings support the favorable benefit–risk profile of secukinumab in real-world clinical practice.

### 3.4 Multivariate correlation analysis of clinical parameters

#### 3.4.1 Comprehensive correlation matrix

**Figure 2:**
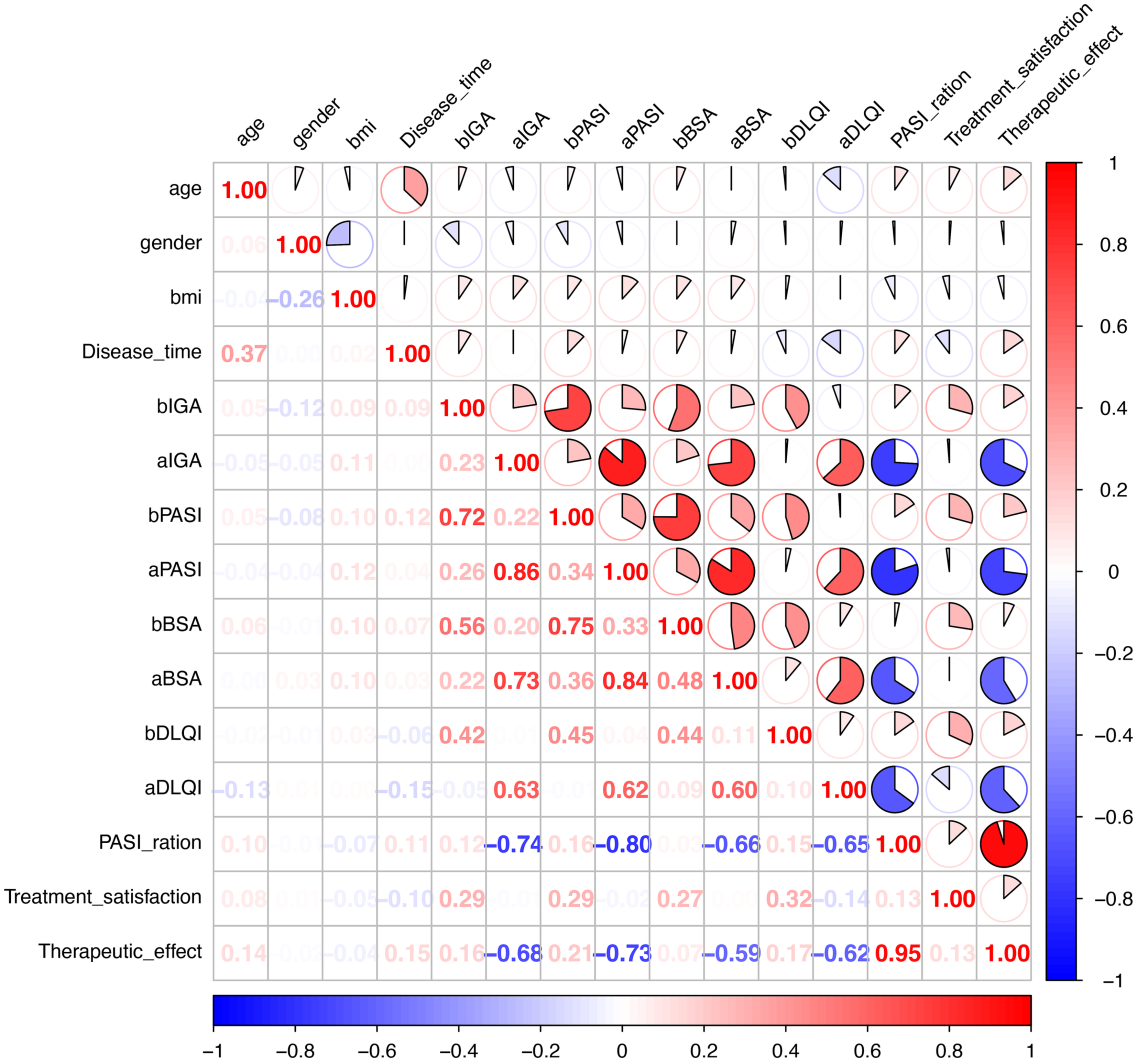
Comprehensive correlation matrix of demographic and clinical variables in psoriatic arthritis patients treated with secukinumab. Correlation coefficients are displayed within cells, with color intensity representing correlation strength (blue = negative correlation; and red = positive correlation). BMI, body mass index; bIGA, baseline investigator’s Global Assessment; aIGA, post-treatment IGA; bPASI, baseline Psoriasis Area and Severity Index; aPASI, post-treatment PASI; bBSA, baseline Body Surface Area; aBSA, post-treatment BSA; bDLQI, baseline Dermatology Life Quality Index; aDLQI, post-treatment DLQI; PASI ratio, proportional PASI improvement.

To systematically evaluate interrelationships among demographic variables, disease severity indices, and treatment response metrics, a comprehensive correlation analysis was performed (Figure 2). Demographic Variable Correlations: Age demonstrated a weak positive correlation with disease duration (*r* = 0.30, *p* < 0.001), consistent with the expected relationship between patient age and cumulative disease exposure. Gender exhibited a weak negative correlation with BMI (*r* = −0.26, *p* < 0.001), reflecting gender-based differences in body composition within the cohort.

Baseline Disease Severity Correlations: Strong intercorrelations were observed among baseline disease severity measures. Baseline IGA demonstrated strong correlations with baseline PASI (*r* = 0.72, *p* < 0.001) and baseline BSA (*r* = 0.56, *p* < 0.001), and a moderate correlation with baseline DLQI (*r* = 0.42, *p* < 0.001). Baseline PASI showed strong associations with baseline BSA (*r* = 0.75, *p* < 0.001) and moderate correlation with baseline DLQI (*r* = 0.45, *p* < 0.001).

Post-Treatment Outcome Correlations: Following treatment, correlations among clinical parameters strengthened considerably. Post-treatment IGA demonstrated very strong correlations with post-treatment PASI (*r* = 0.86, *p* < 0.001), post-treatment BSA (*r* = 0.73, *p* < 0.001), and strong correlation with post-treatment DLQI (*r* = 0.63, *p* < 0.001). Post-treatment PASI exhibited very strong association with post-treatment BSA (*r* = 0.84, *p* < 0.001) and strong correlation with post-treatment DLQI (*r* = 0.62, *p* < 0.001).

Treatment Response Metric Correlations: The PASI ratio, reflecting proportional improvement from baseline, demonstrated strong inverse correlations with all post-treatment severity measures: post-treatment IGA (*r* = −0.74, *p* < 0.001), post-treatment PASI (*r* = −0.80, *p* < 0.001), post-treatment BSA (*r* = −0.66, *p* < 0.001), and post-treatment DLQI (*r* = −0.65, *p* < 0.001). Overall treatment efficacy exhibited a very strong positive correlation with PASI ratio (*r* = 0.95, *p* < 0.001) and strong inverse associations with post-treatment indices, including post-treatment IGA (*r* = −0.68, *p* < 0.001), post-treatment PASI (*r* = −0.73, *p* < 0.001), post-treatment BSA (*r* = −0.59, *p* < 0.001), and post-treatment DLQI (*r* = −0.62, *p* < 0.001).

#### 3.4.2 Post-treatment intermetric relationships

**Figure 3:**
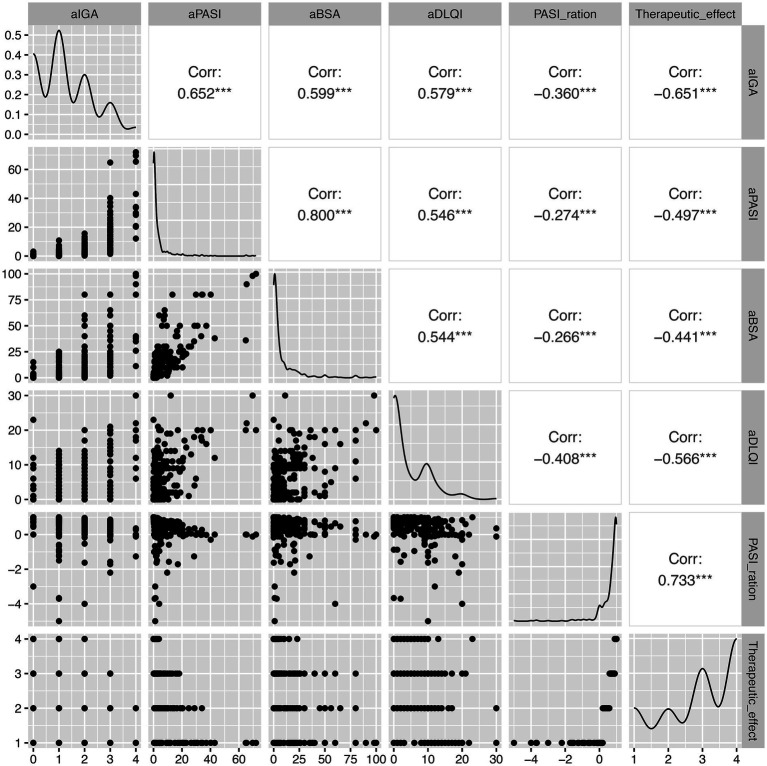
Post-treatment intermetric correlation analysis. Scatterplot matrix with correlation coefficients and linear regression lines illustrating relationships among post-treatment clinical parameters. aIGA, post-treatment Investigator’s Global Assessment; aPASI, post-treatment Psoriasis Area and Severity Index; aBSA, post-treatment Body Surface Area; aDLQI, post-treatment Dermatology Life Quality Index. ***p < 0.001.

Focused analysis of post-treatment outcome correlations (Figure 3) further elucidated the multidimensional nature of treatment response. Post-treatment DLQI exhibited moderate-to-strong correlations with post-treatment IGA (*r* = 0.579, *p* < 0.001), post-treatment PASI (*r* = 0.546, *p* < 0.001), and post-treatment BSA (*r* = 0.544, *p* < 0.001), confirming a consistent alignment between residual clinical disease activity and quality of life impairment. Post-treatment BSA demonstrated strong associations with post-treatment IGA (*r* = 0.599, *p* < 0.001) and very strong correlation with post-treatment PASI (*r* = 0.800, *p* < 0.001). Post-treatment PASI showed strong correlation with post-treatment IGA (*r* = 0.652, *p* < 0.001). The robust correlation between overall treatment efficacy and PASI ratio (*r* = 0.95, *p* < 0.001) underscores the utility of PASI-based metrics as primary efficacy endpoints. The inverse relationships between treatment efficacy and post-treatment severity scores across all domains (IGA, PASI, BSA, and DLQI) highlight the clinical improvements across objective clinician-assessed and subjective patient-reported outcomes.

These findings collectively confirm that secukinumab therapy achieves multidimensional efficacy in PsA, with cutaneous symptom resolution (reflected by BSA and PASI reductions) paralleling clinician-assessed global improvement (IGA) and patient-reported quality of life enhancement (DLQI).

### 3.5 Development and validation of a predictive nomogram for treatment response

**Figure 4:**
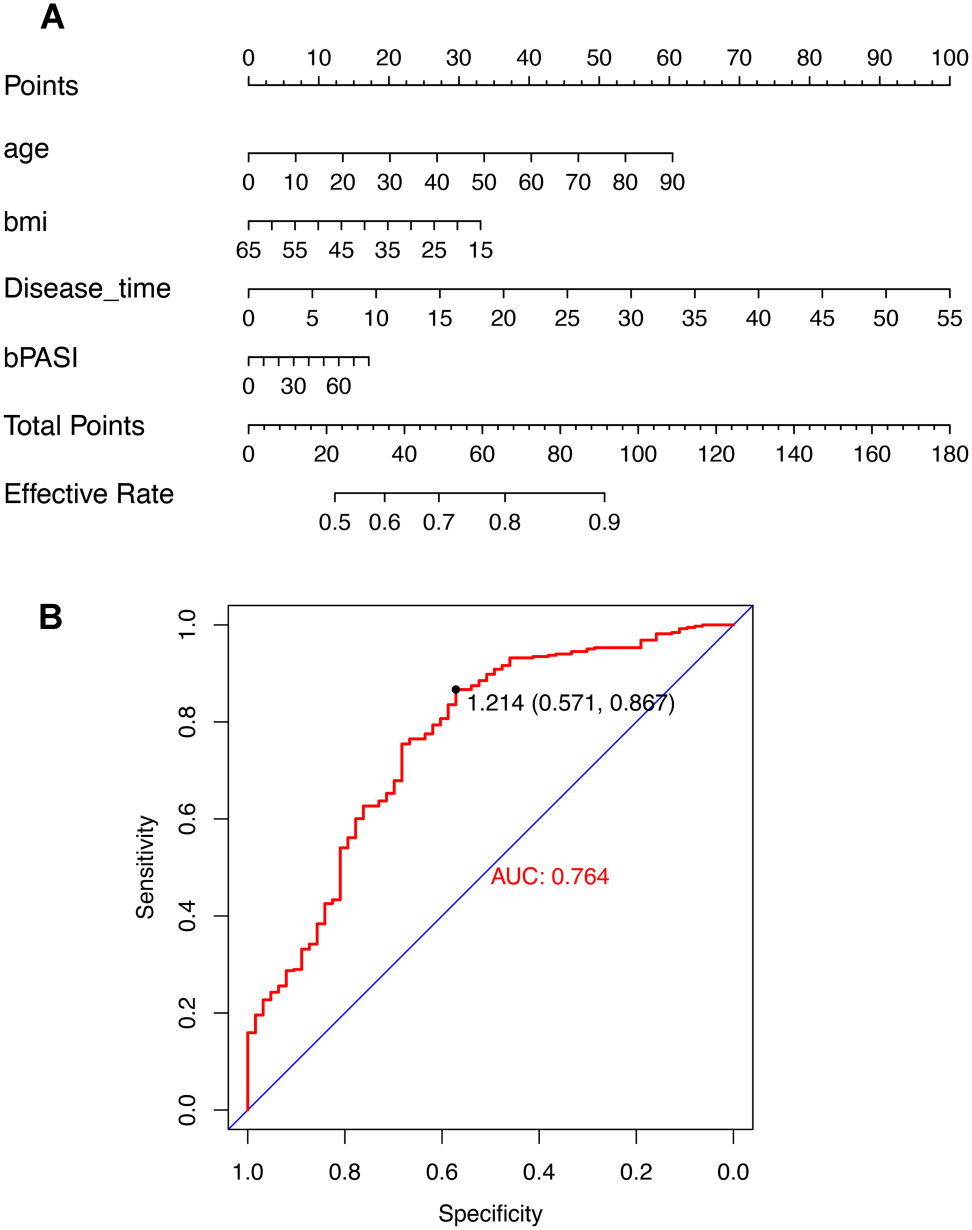
Predictive nomogram for secukinumab treatment efficacy and model validation. **(A)** Nomogram for predicting treatment response to secukinumab in psoriatic arthritis. To calculate the predicted probability of treatment response, locate the patient’s value for each predictor on the corresponding axis, draw a vertical line to the “Points” axis to determine the points for that predictor, sum the points for all predictors, and locate this total on the “Total Points” axis to determine the corresponding predicted probability. **(B)** Receiver operating characteristic (ROC) curve demonstrating model discriminative performance. The area under the curve (AUC) was 0.764. BMI, Body mass index; bPASI, baseline Psoriasis Area and Severity Index.

To develop a clinically applicable tool for predicting secukinumab treatment response, a multivariate regression model was constructed using forward stepwise variable selection. Candidate predictor variables included baseline demographic characteristics (age, gender, and BMI), disease-related factors (disease duration and family history), baseline disease severity indices (PASI, BSA, IGA, and DLQI), and treatment history (prior biologic exposure and pretreatment satisfaction). The final model retained the following significant predictors of treatment efficacy: baseline PASI score, disease duration, patient age, baseline BSA, and BMI. These variables collectively demonstrated independent predictive value for secukinumab treatment response. A nomogram was constructed to provide a graphical representation of the predictive model, facilitating clinical application and individualized treatment response prediction (Figure 4A). The nomogram assigns point values to each predictor variable based on its regression coefficient, allowing clinicians to calculate a total point score for individual patients by summing the points corresponding to their baseline characteristics. The total points can then be mapped to the predicted probability of achieving treatment response. In clinical application, higher baseline PASI scores, longer disease duration, lower BMI, and older age were associated with higher predicted treatment efficacy. This nomogram provides a practical tool for clinicians to identify patients most likely to achieve favorable responses to secukinumab therapy.

Internal validation of the predictive model was performed using 10-fold cross-validation. Discriminative performance was assessed by receiver operating characteristic (ROC) analysis, which demonstrated an area under the curve (AUC) of 0.764 (95% CI: 0.712–0.816; Figure 4B). According to established interpretation criteria, this AUC value indicates fair-to-good discriminative ability, suggesting that the model can adequately distinguish between patients who will and will not achieve treatment response.

## 4 Discussion

The present study provides real-world evidence on the short-term effectiveness and safety of secukinumab in a large cohort of Chinese PsA patients. Importantly, because this was a dermatology-centered retrospective analysis, the outcomes evaluated in this study primarily reflect cutaneous disease severity and dermatology-specific quality of life, rather than the full multidomain spectrum of PsA activity. Within this context, secukinumab was associated with robust improvement across objective skin measures (PASI, BSA, and IGA) and patient-reported dermatology-specific quality of life (DLQI), while also showing a favorable short-term safety profile. In addition, this study developed an internally validated nomogram that may help estimate the probability of short-term skin response based on baseline clinical characteristics.

### 4.1 Efficacy of secukinumab in real-world clinical practice

Our study demonstrated substantial improvements in disease severity following secukinumab treatment, with mean PASI scores decreasing by 72.0% (from 14.2 to 3.98) and BSA involvement reducing by 67.9% (from 25.7 to 8.26%). These findings are consistent with, and in some respects exceed, the efficacy outcomes reported in pivotal randomized controlled trials. In the FUTURE 2 trial, secukinumab 300 mg achieved PASI 75 response rates of 48.2% at week 24 in PsA patients (11), while pooled analyses from the FUTURE program demonstrated sustained skin clearance through 52 weeks of treatment (17). The real-world data extend these observations by confirming that the efficacy demonstrated in controlled trial settings translates effectively to routine clinical practice in a Chinese patient population.

The magnitude of DLQI improvement observed in the study cohort (mean reduction of 7.5 points, from 11.9 to 4.41) is particularly noteworthy. According to established DLQI banding interpretations (3, 18), this represents a clinically meaningful transition from “very large effect” to “small effect” on quality of life. This improvement exceeds the minimal clinically important difference (MCID) of 4 points established for DLQI in psoriasis populations (19), underscoring the substantial patient-perceived benefit of secukinumab therapy. These findings align with previous reports highlighting the importance of IL-17A inhibition in addressing both cutaneous manifestations and the broader psychosocial burden of psoriatic disease (20, 21).

Compared with other biologic agents used in PsA management, secukinumab demonstrates competitive efficacy. Meta-analyses comparing IL-17 inhibitors with TNF inhibitors and IL-12/23 inhibitors have shown comparable or superior skin clearance rates with IL-17A blockade (22, 23). The real-world findings support these comparative data and suggest that secukinumab represents an effective therapeutic option for Chinese PsA patients, a population that has been underrepresented in international clinical trials.

### 4.2 Enhanced alignment between objective severity and dermatologic quality of life

A notable contribution of this study is the characterization of how the relationship between objective disease severity and patient-reported quality of life evolves following effective treatment. The study observed that the correlation between PASI and DLQI strengthened substantially after secukinumab therapy (*R*^2^ increasing from 0.34 to 0.52). This phenomenon is described as an enhanced clinico-dermatologic QOL alignment.

It is important to note that DLQI captures the dermatology-specific impact on daily life rather than validated psychological constructs such as depression or anxiety. The strengthened post-treatment correlation therefore reflects improved alignment between clinician-assessed skin severity and patient-perceived dermatologic disease impact, rather than a demonstrated effect on psychological well-being per se. Assessment of psychological outcomes would require validated psychiatric instruments such as the Hospital Anxiety and Depression Scale (HADS) or Patient Health Questionnaire (PHQ-9), which were not included in this study.

It should be emphasized that the findings of this study are derived exclusively from PsA patients and may not be generalizable to patients with plaque psoriasis without musculoskeletal involvement. In PsA, quality-of-life impairment is multifactorial, encompassing joint pain, functional disability, enthesitis, dactylitis, fatigue, and psychological comorbidities beyond what is captured by DLQI. The baseline PASI–DLQI correlation (*R*^2^ = 0.34) observed in the study cohort likely reflects this multidomain disease burden — a substantial proportion of baseline QOL impairment is attributable to non-cutaneous factors not measured by PASI. In psoriasis-only populations, where skin disease constitutes the predominant disease burden, the baseline PASI–DLQI relationship and its post-treatment trajectory may differ fundamentally.

### 4.3 Correlation analysis and clinical implications

The comprehensive correlation analysis revealed several clinically relevant patterns. The strong intercorrelations among baseline disease severity measures (IGA–PASI: *r* = 0.72; PASI–BSA: *r* = 0.75) confirm the internal consistency of these assessment tools and support their interchangeable use in clinical practice and research settings. The moderate correlations between objective severity measures and baseline DLQI (*r* = 0.42–0.45) are consistent with prior literature demonstrating that quality of life impairment is related to, but not entirely determined by, clinical disease severity (24, 25). Notably, correlations among clinical parameters strengthened following treatment, with post-treatment IGA-PASI correlation reaching *r* = 0.86 and post-treatment PASI–BSA correlation reaching *r* = 0.84. This convergence of outcome measures following treatment suggests that secukinumab is associated with a coherent therapeutic response across multiple disease dimensions, rather than differentially affecting individual parameters. Such consistency is reassuring for clinical decision-making and supports the use of any validated severity measure for treatment monitoring. The very strong correlation between PASI ratio and overall treatment efficacy (*r* = 0.95) validates the use of PASI-based response criteria (PASI 50, PASI 75, and PASI 90) as primary efficacy endpoints. The inverse correlations between treatment efficacy and all post-treatment severity measures (*r* = −0.59 to −0.73) further confirm that therapeutic success, as defined by PASI improvement, translates consistently to improvements across clinician-assessed (IGA) and patient-reported (DLQI) domains.

### 4.4 Predictive nomogram for personalized treatment selection

The development of a predictive nomogram represents a practical contribution to personalized medicine in PsA management. Our model identified baseline PASI score, disease duration, patient age, baseline BSA, and BMI as independent predictors of secukinumab treatment response. The model achieved an AUC of 0.764, indicating fair-to-good discriminative ability that is clinically meaningful for informing treatment decisions. The finding that higher baseline PASI predicts favorable treatment response warrants careful contextualization within the broader literature. While the study results align with several studies demonstrating greater absolute and relative PASI improvements in patients with more severe baseline disease (26, 27), this relationship is not universally consistent across real-world cohorts. Yıldırım et al. reported that higher baseline PASI may be associated with a reduced likelihood of achieving stringent complete clearance endpoints such as PASI 100 in psoriasis patients treated with biologics (28). This apparent discrepancy likely reflects several factors. First, the treatment response definition was based on proportional PASI improvement (PASI ratio) rather than an absolute clearance threshold, and patients with higher baseline scores have greater mathematical capacity for proportional improvement. Second, population differences between PsA and psoriasis-only cohorts may influence the relationship, as PsA patients may exhibit different inflammatory phenotypes and treatment response patterns. Third, variations in prior biologic exposure rates (32.5% in this study cohort), ethnic composition, disease duration distributions, and concomitant medication use across studies may modulate the baseline severity–response relationship. Fourth, the specific biologic agent and mechanism of action under investigation may interact differently with baseline disease severity. This study acknowledge that the positive predictive value of baseline PASI in the model should be interpreted within the context of specific response definition and population characteristics, and may not generalize to settings using stringent absolute clearance endpoints.

The associations of disease duration and age with treatment response warrant careful interpretation. Longer disease duration was associated with better predicted efficacy in this model, which may reflect patient selection effects or accumulated disease burden that responds favorably to effective intervention. The relationship between age and treatment response may be mediated by immunological changes associated with aging or by differences in disease phenotype across age groups (29). The inverse association between BMI and treatment efficacy aligns with emerging evidence that obesity may attenuate response to biologic therapies in psoriatic disease (30). Proposed mechanisms include altered drug pharmacokinetics in obese patients, adipose tissue-derived inflammatory mediators that may counteract biologic efficacy, and metabolic comorbidities that independently influence disease activity (31). These findings support recommendations for weight management as an adjunctive strategy in PsA patients receiving biologic therapy.

From a practical standpoint, the nomogram provides substantial clinical utility by translating complex statistical modeling into an accessible visual tool. Clinicians can use easily obtainable baseline characteristics to predict the probability of a favorable relative skin response. This facilitates shared decision-making, helps manage patient expectations, and may identify patients who require closer monitoring or adjunctive therapies when their predicted response is suboptimal.

### 4.5 Safety considerations

The low TRAE rate observed in this study should be interpreted cautiously, as safety data were derived from retrospective chart review, limited to physician-attributed treatment-related events, and assessed over a relatively short follow-up period. Mild transient adverse events may have been under captured in routine practice. Accordingly, the favorable short-term safety profile observed in this study cohort should be interpreted with caution. Physician-documented treatment-related adverse events were reported in only 0.90% of patients, which is lower than rates described in pivotal trials and many real-world studies. Several factors may explain this difference. First, the present analysis was based on retrospective chart review and captured only physician-attributed treatment-related adverse events, rather than all adverse events occurring during exposure. Second, the follow-up period was relatively short (12–16 weeks), limiting the opportunity to detect delayed or cumulative events. Third, mild, transient, or self-limited events may have been under documented in routine clinical practice. Population-level differences, including ethnic and practice-pattern variation, may also contribute, although such explanations should be interpreted cautiously. Importantly, the absence of serious adverse events in this cohort should not be interpreted as evidence of long-term safety superiority. Rather, these findings suggest that secukinumab demonstrated a favorable short-term tolerability profile in this real-world PsA cohort under routine clinical care conditions.

### 4.6 Strengths and limitations

This study has several notable strengths. First, the large sample size (*n* = 446) provides robust statistical power for efficacy analyses and correlation assessments. Second, the real-world design captures treatment outcomes in routine clinical practice, enhancing external validity and generalizability compared with controlled trial settings. Third, the comprehensive assessment of multiple validated outcome measures allows multidimensional characterization of treatment response. Fourth, the novel analysis of PASI–DLQI relationship dynamics provides new insights into the interplay between objective and subjective disease outcomes. Finally, the predictive nomogram offers practical clinical utility for personalized treatment planning.

Several limitations must be acknowledged. First, as a PsA-specific study, our findings regarding the PASI–DLQI relationship and predictive model cannot be directly generalized to psoriasis patients without musculoskeletal disease, given the additional disease dimensions in PsA that independently influence quality-of-life outcomes. Our cohort exclusively consisted of patients diagnosed with PsA according to CASPAR criteria. Because PsA is a systemic disease where QOL is heavily influenced by joint involvement, these findings regarding the PASI–DLQI relationship cannot be broadly generalized to psoriatic patients without musculoskeletal disease. Second, due to the dermatological focus of the registry, this study lacked detailed rheumatological parameters, such as ACR response criteria, enthesitis, and dactylitis assessments, limiting the conclusions primarily to cutaneous outcomes. Third, the retrospective observational design precludes causal inference; therefore, associations between variables should be interpreted descriptively rather than mechanistically. Finally, while internally validated, the predictive nomogram lacks external validation. Future studies in independent, multicenter cohorts are required to confirm its predictive reliability before widespread clinical application.

### 4.7 Future directions

Several avenues for future research emerge from these findings. Longitudinal studies with extended follow-up are needed to characterize the durability of treatment response and the long-term trajectory of alignment between clinical and quality-of-life outcomes. Comparative effectiveness research directly comparing secukinumab with other biologic agents in Chinese PsA patients would inform optimal treatment selection. External validation of the predictive nomogram in independent cohorts, including non-Chinese populations, would establish its broader applicability. Investigation of the biological mechanisms underlying alignment between clinical and quality-of-life outcomes, potentially through biomarker studies correlating inflammatory mediators with quality-of-life trajectories, may provide mechanistic insights with therapeutic implications. Finally, integration of joint-specific outcomes and imaging parameters into future predictive models would enhance their comprehensiveness for PsA, a disease characterized by both skin and musculoskeletal manifestations.

This study does not include immune biomarkers, cytokine profiling, or mechanistic analyses. The observed correlational patterns therefore represent statistical associations between clinical endpoints and cannot be interpreted as evidence of specific immunological mechanisms. Future studies incorporating inflammatory mediator measurements, tissue biopsy analyses, and validated psychological instruments are needed to elucidate the biological basis of the observed clinical patterns.

## 5 Conclusion

In conclusion, this large real-world study demonstrates that secukinumab provides robust efficacy across objective disease severity measures and patient-reported quality of life outcomes in Chinese PsA patients, with an excellent safety profile. The novel observation of alignment between clinical and quality-of-life outcomes—enhanced concordance between clinical improvement and quality of life gains following effective treatment—provides new insights into the multidimensional nature of therapeutic response and supports treat-to-target strategies pursuing stringent efficacy endpoints. The predictive nomogram offers a practical tool for personalized treatment planning, though external validation is warranted. These findings contribute to the growing evidence base supporting IL-17A inhibition as an effective therapeutic strategy for PsA and provide a framework for optimizing treatment outcomes through individualized patient selection and comprehensive outcome assessment.

## Data Availability

The original contributions presented in the study are included in the article/supplementary material, further inquiries can be directed to the corresponding author.
